# Data-driven analysis of immune infiltrate in a large cohort of breast cancer and its association with disease progression, ER activity, and genomic complexity

**DOI:** 10.18632/oncotarget.19078

**Published:** 2017-07-07

**Authors:** Ruth Dannenfelser, Marianne Nome, Andliena Tahiri, Josie Ursini-Siegel, Hans Kristian Moen Vollan, Vilde D. Haakensen, Åslaug Helland, Bjørn Naume, Carlos Caldas, Anne-Lise Børresen-Dale, Vessela N. Kristensen, Olga G. Troyanskaya

**Affiliations:** ^1^ Department of Computer Science, Princeton University, Princeton, New Jersey, United States of America; ^2^ Lewis-Sigler Institute for Integrative Genomics, Princeton University, Princeton, New Jersey, United States of America; ^3^ Institute for Clinical Medicine, Faculty of Medicine, University of Oslo, Oslo, Norway; ^4^ Department of Clinical Molecular Oncology, Division of Medicine, Akershus University Hospital, Ahus, Norway; ^5^ Lady Davis Institute for Medical Research, McGill University, Montreal, Quebec, Canada; ^6^ Cancer Research UK Cambridge Institute, University of Cambridge, Cambridge, United Kingdom; ^7^ Department of Genetics, Institute for Cancer Research, Oslo University Hospital, The Norwegian Radium Hospital, Oslo, Norway; ^8^ Department of Oncology, Division for Surgery, Cancer, and Transplantation, Oslo University Hospital, The Norwegian Radium Hospital, Oslo, Norway; ^9^ Flatiron Institute, Simons Foundation, New York, New York, United States of America

**Keywords:** breast cancer, immune profiling, lymphocyte infiltration, normal breast tissue, immune infiltration

## Abstract

The tumor microenvironment is now widely recognized for its role in tumor progression, treatment response, and clinical outcome. The intratumoral immunological landscape, in particular, has been shown to exert both pro-tumorigenic and anti-tumorigenic effects. Identifying immunologically active or silent tumors may be an important indication for administration of therapy, and detecting early infiltration patterns may uncover factors that contribute to early risk. Thus far, direct detailed studies of the cell composition of tumor infiltration have been limited; with some studies giving approximate quantifications using immunohistochemistry and other small studies obtaining detailed measurements by isolating cells from excised tumors and sorting them using flow cytometry. Herein we utilize a machine learning based approach to identify lymphocyte markers with which we can quantify the presence of B cells, cytotoxic T-lymphocytes, T-helper 1, and T-helper 2 cells in any gene expression data set and apply it to studies of breast tissue. By leveraging over 2,100 samples from existing large scale studies, we are able to find an inherent cell heterogeneity in clinically characterized immune infiltrates, a strong link between estrogen receptor activity and infiltration in normal and tumor tissues, changes with genomic complexity, and identify characteristic differences in lymphocyte expression among molecular groupings. With our extendable methodology for capturing cell type specific signal we systematically studied immune infiltration in breast cancer, finding an inverse correlation between beneficial lymphocyte infiltration and estrogen receptor activity in normal breast tissue and reduced infiltration in estrogen receptor negative tumors with high genomic complexity.

## INTRODUCTION

Cancer cells develop in the extracellular matrix (ECM) surrounded by a variety of non-malignant cells, such as fibroblasts, vascular cells, leukocytes, and bioactive substances such as chemokines and cytokines. Together these cells and substances of the host form an environment conducive to carcinogenesis [1–3]. Leukocytes, once thought to be purely beneficial, are now recognized for their functions in tumor promotion as well as inhibition. Many cells of the myeloid lineage contribute to tumor proliferation and cancer development across tumor types. More specifically, tumor associated M2 macrophages, neutrophils, mast cells, immature myeloid cells, and monocytes have been widely shown to support cancer progression through the secretion of growth factors, cytokines, and proteases that promote remodeling of the ECM [4], genomic instability [5–6], angiogenesis [7–11], and suppression of beneficial immune responses [12]. Lymphocytes, while generally having a positive effect, exert pro-tumor or anti-tumor functions in a tissue and cancer specific manner [13]. This effect is largely due to their plasticity [14–15].

There is substantial variability in the number and types of infiltrating lymphocytes in breast tumors across individuals. Recent efforts to profile this landscape have found both of these properties to be indicative of outcome and response to chemotherapy [16–20]. Furthermore, there has been specific interest in finding the component of tumor infiltrating lymphocytes (TILs) that are specifically recruited in the attempt to control tumor growth. These will be of particular importance in the era of immune checkpoint inhibitors such as PD1.

Thus far the established critical factor of the lymphocyte anti-tumor response is the presence of CD8^+^ cytotoxic T-cells (CTL), which contribute directly to apoptosis through the secretion of cytotoxins. Numerous breast cancer studies have documented their association with good prognosis and long-term survival [12, 18, 21–23]. T-helper 1 (Th1) cells are also widely thought to contribute to tumor clearance, through the production of interferon gamma (IFNG) which helps curb proliferation, slow angiogenesis, enhance M1 macrophages’ tumoricidal ability, and aid in CTL efficacy through the expression of the major histocompatibility complex class I [12, 24]. The exact role of T-helper 2 (Th2) cells in breast cancer is less clear, but generally they are thought to be pro-tumorgenic. In mouse models, Th2 driven expression of IL13 and IL4 contribute to tumor progression and metastasis [12, 25–27]. Additionally, IL4 increases leukocyte recruitment and the promotion of epidermal growth factor by M2 macrophages [28]. However, IL13 has been shown to reduce breast cancer recurrence [25]. The function of B cell infiltration is poorly understood, with some studies highlighting the importance of B cells in good outcome [29–30], some in poor outcome [12], and others deeming B cell infiltration irrelevant [28]. Along with the recognition of the critical role played by the immune system in oncogenesis, tumor progression, and response to therapy, increasing attention has been drawn by the potential prognostic and predictive role of the immune infiltrate in this setting.

Traditional studies profile lymphocyte infiltration utilizing immunohistochemical staining or, less commonly, single cell isolation followed by flow cytometry to assess the composition and abundance of lymphocytes within a tumor sample. Staining based approaches are semi-quantitative at best, while flow based approaches are labor intensive and difficult to perform on a large scale. Additionally, both methods require access to and destruction of a portion of the tumor sample. Herein we propose an alternative *in silico* approach to examine breast tumor infiltration by four major lymphocyte cell types: B cells, CTL, Th1, and Th2 cells. We make use of the fact that gene expression assays of tumor samples capture signal that is representative of tumor cells as well as their microenvironment. Our method is robust, correlates well with experimental measures of immune infiltration, and allows us to retroactively profile lymphocyte abundance in existing breast tumor expression data without a priori knowledge of sample composition. With this approach we show changes in infiltrate levels in estrogen receptor negative normal breast tissue, reproducible patterns of immune activity across molecular groupings, and examine the impact these levels have on patient survival and treatment response. Through this analysis we establish critical links between the molecular features of breast tissue and immune infiltration and demonstrate how our marker set approach can be effectively applied towards this end.

## RESULTS

### *In silico* immune profiling derived from a compendium of 2,171 human samples of cultured and primary human tissues and treatments

**Figure 1:**
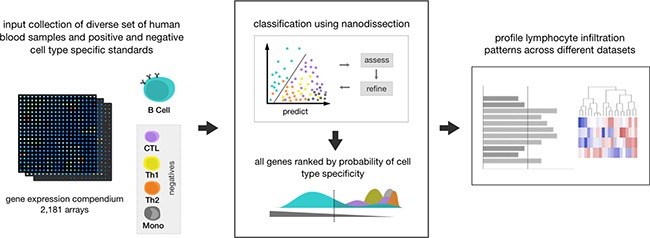
Method overview This diagram shows the process of running our method on one cell type, B cells. First all input data, consisting of over 2,000 human blood microarray samples taken in different conditions and different degrees of resolution, and positive and negative training standards, are assembled. In this example, B cell genes from Gene2MeSH are used as a positive standard and all other lymphocyte cell types (CTL, Th1, Th2, monocytes) in Gene2MeSH are used as negatives. Using the input, nanodissection finds a path that separates the positive training class from the negative ones in the space of the compendium, iteratively improving classification by selecting the best set of standards. Using the classification boundary, all genes assayed are then ranked by their probability to be cell type specific, in this case B cell specific. We then use the top 100 most probable cell type specific genes to estimate lymphocyte infiltration levels in breast cancer datasets.

We used an *in silico* method to find genes that are preferentially expressed in four lymphocyte cell types: B cells, CD8^+^ T cells (CTL), CD4^+^ T helper 1 (Th1), and CD4^+^ T helper 2 (Th2) cells (Figure 1). For each cell type we expanded a set of literature mined marker genes into a robust set of cell type specific genes using a large compendium of human blood expression data and the nanodissection method (http://nano.princeton.edu) we developed previously for computationally predicting cell-type specific genes [31]. Nanodissection uses an iterative machine learning framework with diverse expression data collections, to identify expression patterns specific to marker genes for a given cell type. We validated this approach previously in systematic computational and experimental evaluations [31]. When applied to the four lymphocyte cell types, (see Materials and Methods) the resulting set of markers enable us to robustly identify immune specific signal (Supplementary Figure 2, Supplementary Figure 3).

**Figure 2:**
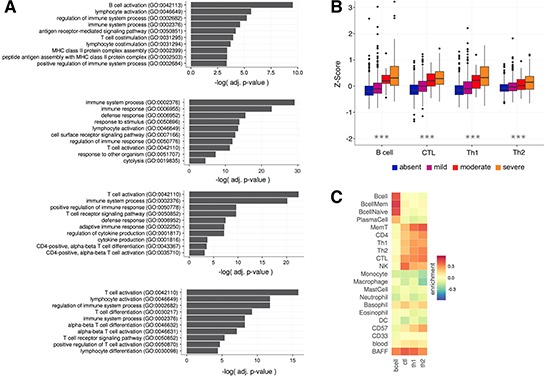
Expression of lymphocyte marker genes correspond with experimentally defined measures of immune infiltration (**A**) GO Biological Process enrichment for each of the lymphocyte marker gene sets. Shown here are some of the relevant significant terms (a full list can be found in Supplementary Table 3). Each marker is significantly enriched for immune related terms that are representative of the role of their corresponding cell type. (**B**) Four categories of immune infiltration (absent, mild, moderate, severe) were defined by H&E staining for the METABRIC cohort [38]. The box plots show that B cell, CTL, Th1, and Th2 marker co-expression correspond with increasing levels of infiltration. All labels were significantly different between the infiltration categories calculated using ANOVA (***p < 1.0e^−5^) (**C**) Average enrichment of marker genes across purified immune cell types from Chtanova et al [34], GSE3982, and GSE1133.

The overall performance of nanodissection, for each lymphocyte cell type, was assessed based on classification performance and the relevance of the resulting marker sets. In five-fold cross validation we achieve AUCs above 0.7 for each nanodissection run: 0.8896, 0.822, 0.732, and 0.722 for B cells, CTL, Th1, and Th2 cells respectively (Supplementary Figure 1). We confirmed the biological relevance of our resulting marker sets by analyzing the functional processes they participate in and the correspondence of our predictions with experimental measures of lymphocyte infiltration. To identify overrepresented pathways, we calculated statistical enrichment for each of the resulting marker gene sets. The top significantly enriched terms are predominantly immune related and are representative of the action of each lymphocyte subset (Figure 2A, Supplementary Table 3), with B cell activation enriched for the B cell marker, cell defense terms such as cytolysis for CTLs, and many T cell activation terms enriched for the T-helper cells.

Furthermore, we examined the relationship between the expression level of the immune markers and experimentally derived immune infiltration categories (absent, mild, moderate, severe) in the METABRIC breast cancer cohort [38]. We calculated a B cell, CTL, Th1, and Th2 infiltration score for each sample by averaging the expression of all genes in each marker. With these scores we observe that B cell (*ANOVA*, *p* = 3.7e^−4^), CTL (*p* < 2.2e^−16^), Th1 (*p* < 2.2e^−16^), and Th2 (*p* < 2.2e^−16^) expression changed significantly across the infiltration categorizations with increases in B cell, CTL, Th1, and Th2 marker expression corresponding with increasing immune infiltration (*Kendall's Tau*, 0.294, 0.290, 0.242, 0.155, Figure 2B). To measure the specificity of our signal we also looked at the enrichment of our marker genes across 185 samples of cell type specific immune expression data, finding that our gene sets are enriched in the corresponding target and related cell types, improving on the specificity of using known standards and T cell surface markers alone (Figure 2C, Supplementary Figure 3).

We also examine the robustness of our signatures to expression data where a subset of our marker genes may not be measured due to platform differences or experimental errors. To test this we randomly removed subsets of genes from each of our markers and recalculated the relationship between the experimentally defined infiltration categories in METABRIC and our predicted B cell, CTL, Th1, and Th2 infiltration scores using Kendall's correlation. With all our signatures we were again significantly able to preserve the infiltration trends up to 50% “lost” genes. Robustness varies only slightly between the cell types, with only the Th2 signature affected with performance degradation around 45% of genes removed (Supplementary Figure 2).

### Lymphocyte infiltration is strongly tied to estrogen receptor status

**Figure 3:**
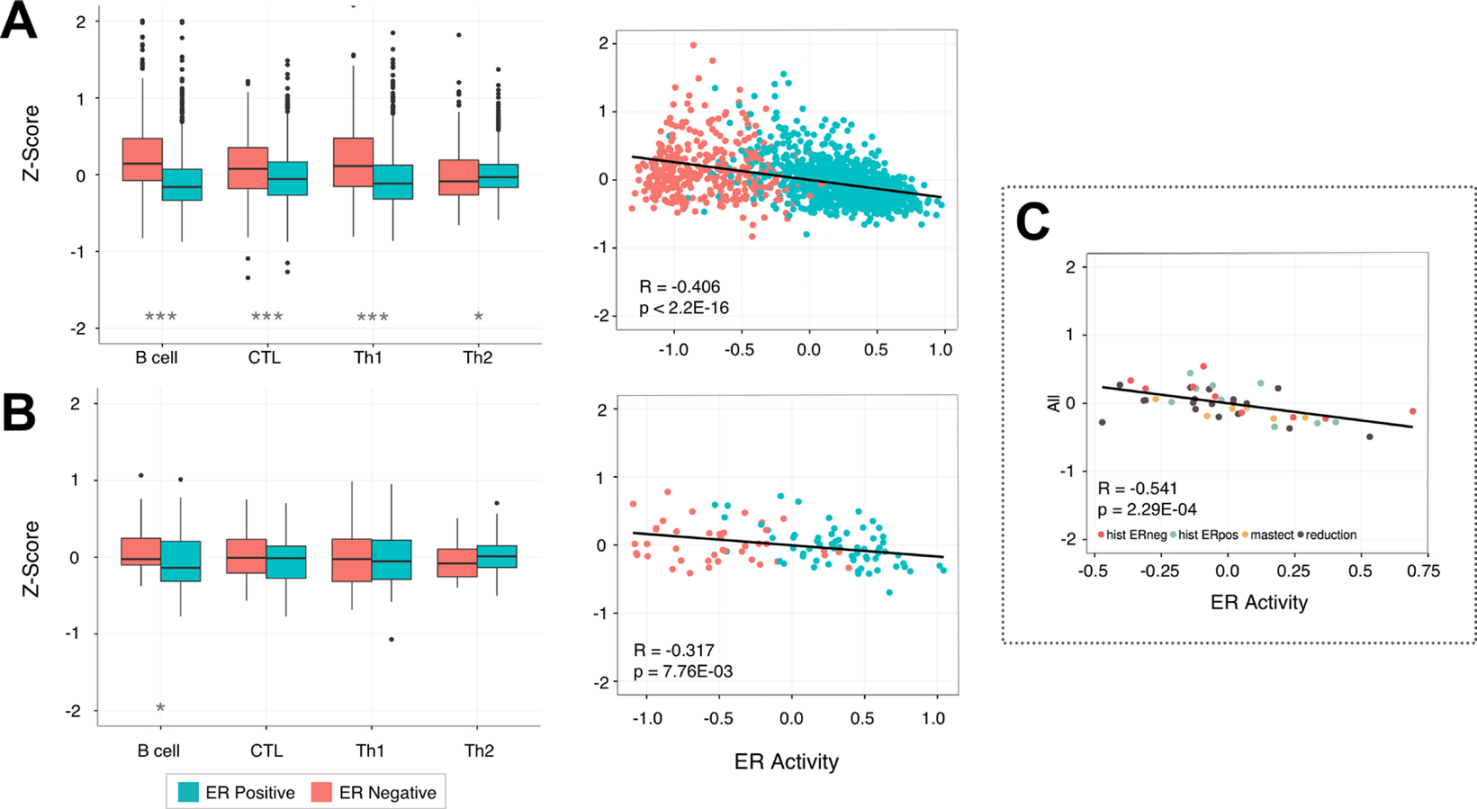
Lymphocyte infiltration is dependent on the activity of the estrogen receptor Box plots show the increased levels of B cell, CTL, Th1, and Th2 infiltration in ER- samples when they are partitioned by estrogen receptor status. The scatterplots show an inverse correlation between the activity of the estrogen receptor and the infiltration of all four lymphocyte subsets in breast tissue. This pattern is reproducible in two separate breast cancer cohorts: METABRIC (**A**) and MicMa (**B**), and is also apparent in a set of normal breast tissue samples taken from adjacent tumor tissue, preventative mastectomy patients, and breast reductions (**C**). *P*-values were calculated using the Wilcoxon rank sum test with *p < 0.05, **p < 0.01, ***p < 0.00001.

At the most basic level breast tumors are divided based on their ability to respond to estrogen. Thus, we examined whether changes in estrogen receptor (ER) status have any effect on lymphocyte infiltration. In this analysis we used two datasets, MicMa (*N* = 108) [39] and the METABRIC (*N* = 1417) cohort [38]. These cohorts together allow us to assess a reasonable number of ER- samples: *N* = 42 and *N* = 340 respectively. After partitioning the samples by ER status we observed a significant increase in the expression levels of all lymphocytes in ER- tumors relative to their ER+ counterparts in METABRIC (*Wilcoxon rank sum test*, Bcell, CTL, Th1: *p* < 1.00e^−7^ and Th2: *p* = 0.023), and B cell in the much smaller MicMa dataset (B cell: *p* = 0.02, CTL: *p* = 0.404, Th1: *p* = 0.833, Th2: 0.057, Figure 3A, 3B). To further characterize this relationship we devised an ER score using the ER marker genes of van't Veer et al. [37] to capture the activity level of the estrogen receptor. For each cohort we observe a significant negative correlation between ER activity and the level of B cell (*Pearson, R* = −0.459, *p* < 2.2e^−16^; *R* = −0.406, *p* = 1.34e^−5^), CTL (*R* = −0.292, *p* < 2.2e^−16^; *R* = −0.343, *p* = 2.83e^−4^), and Th1 (*R* = −0.401, *p* < 2.2e^−16^; *R* = −0.247, *p* = 0.023) infiltration as well as the total level of all four lymphocytes (*R* = −0.406, *p* < 2.2e^−16^; *R* = −0.317, *p* = 5.87e^−8^, Figure 3A, 3B) further supporting our claim. We then extended this analysis to normal tissue, profiling another dataset containing reduction mammoplasties (*N* = 18), adjacent normal tissue from ER+ and ER- patients (*N* = 18), and prophylactic mastectomies (*N* = 6) [40], and found a gradient in the levels of immune infiltration, with a few samples marked by high lymphocyte infiltration. Interestingly, the level of infiltration in these samples can also be explained by estrogen receptor status, as opposed to the sample source. Again using an ER activity score as a proxy for ER status we observed a negative correlation between ER activity and total infiltration (*Pearson, R* = −0.541, *p* = 2.29e^−4^, Figure 3C), suggesting an early link between immune infiltration and ER status that is also preserved in benign samples (Supplementary Figure 4).

### Lymphocyte infiltration is predictive of survival in ER- tumors

**Figure 4:**
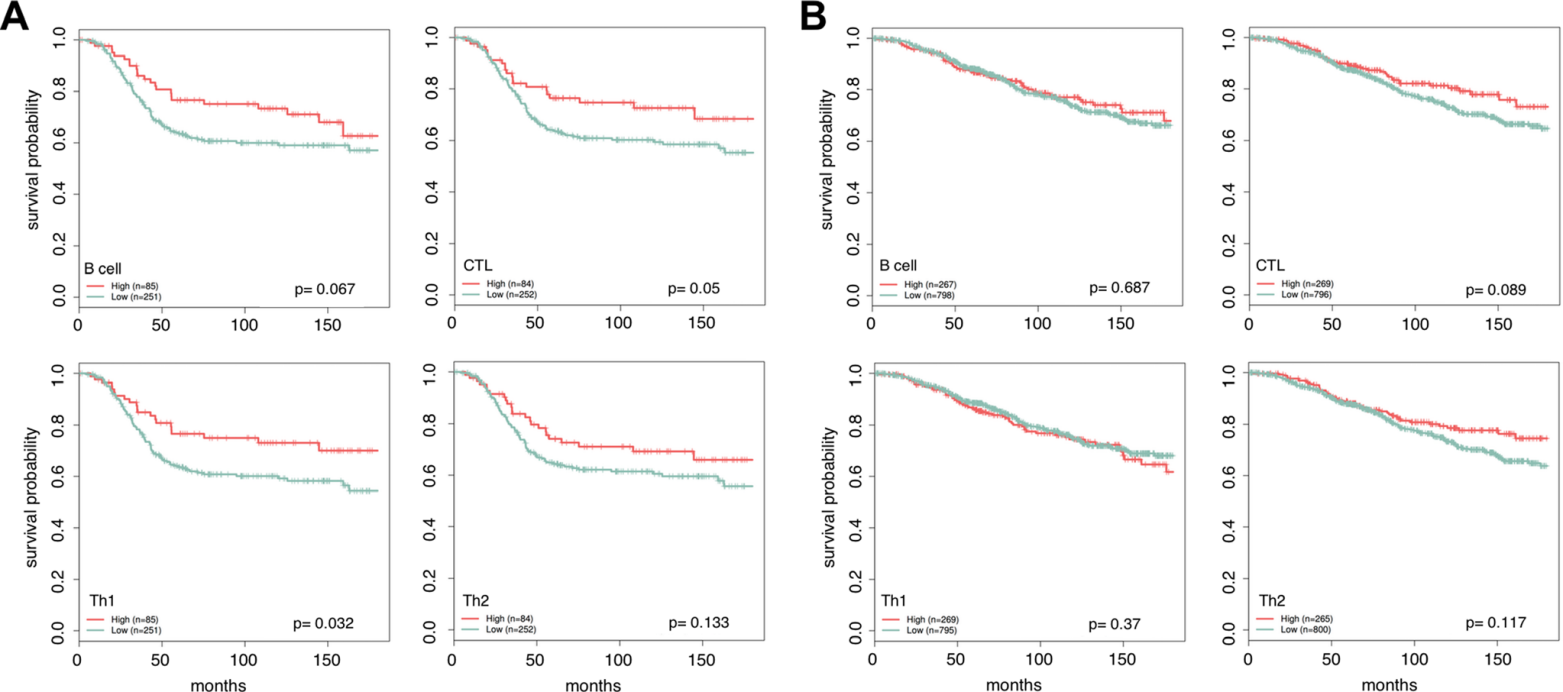
Patients with ER- tumors and high levels of Th1 and CTL infiltration are more likely to have a positive outcome For each lymphocyte marker, patients are partitioned into high and low levels using quartiles. Patients with lymphocyte scores in the upper quartile are marked as high (red) and the remaining quartiles are grouped together and labeled low (green). Increased lymphocyte infiltration is associated with better long term survival in ER- tumors (**A**), and has little effect in ER+ tumors (**B**). The most extreme difference is seen in patients with high Th1 and CTL infiltration vs. low. Reported *p*-values were calculated using the log rank test.

With our markers we can examine both a general and detailed picture of the effects of lymphocyte infiltration on survival. We grouped patients into low (bottom three quartiles) and high (upper quartile) infiltration classes for the overall lymphocyte expression score and separately for the each of the lymphocyte cell types. We then partitioned samples from the METABRIC cohort based on ER status and examined the effects of expression of our lymphocyte markers on patient survival times using univariate Kaplan Meier analysis [41]. Comparing the low and high classes, we see that the total expression of lymphocytes is an important prognostic factor only in ER- tumors (Figure 4). At higher resolution, we find that ER- samples with an improved outcome tend to have higher levels of lymphocyte infiltration, with the levels of CTL and Th1 infiltration playing a significant role (*log rank*, *p* = 0.067, *p* = 0.05, *p* = 0.032, *p* = 0.132). This result is consistent with several previous studies [18, 21, 23, 42] that have observed an improved treatment response and better prognosis in ER- tumors with increased levels of CTL infiltration.

### Unique immune infiltration patterns across breast cancer subtypes

**Figure 5:**
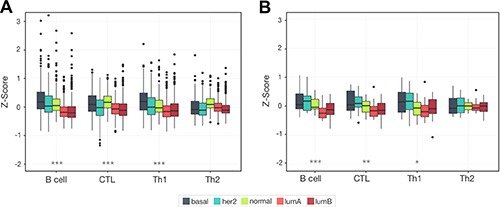
Unique lymphocyte infiltration patterns are seen for each breast cancer subtype Box plots show the distribution of lymphocyte infiltration scores for each subtype. We see a significant reduction in the average level of lymphocyte infiltration in the luminal subtypes (***p < 1.0e^−5^), with the highest level of infiltration in basal and normal-like subtypes. This pattern is likely reflective of the strong link between infiltration and ER negativity and is reproducible across two cohorts METABRIC (**A**) and MicMa (**B**).

Different breast cancer subtypes are commonly referenced as disparate diseases. As such we expect them to have very different and reproducible patterns of lymphocyte expression. Examining our B cell, CTL, Th1, and Th2 scores across samples grouped by subtype we saw that very phenomenon in both tumor cohorts: METABRIC and MicMa (Figure 5). Overall lymphocyte infiltration showed the most pronounced difference between the luminal subtypes and the basal, HER2, and normal-like subtypes, recapitulating the patterns observed with ER status. Elevated infiltration in the predominantly ER- subtypes was significantly increased compared to the luminal subtypes for B cell, CTL, and Th1 in METABRIC (*Wilcoxon rank sum test*, B cell, CTL, and Th1: *p* < 2.2e^−16^, Th2: *p* = 0.067) and MicMa (B cell: *p* = 3.56e^−6^, CTL: *p* = 2.02e^−4^, Th1: *p* = 0.013, Th2: *p* = 0.635).

### Genomic instability is associated with a reduction in beneficial immune responses

**Figure 6:**
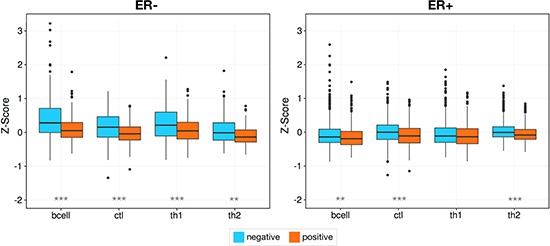
Genomic instability is associated with a reduction in infiltration Higher levels of infiltration are seen in samples with less genomic instability as measured by CAAI status, with a markedly stronger effect in ER- tumors. Asterisks denote Wilcoxon rank sum test *p*-values *p < 0.05, **p < 0.01, ***p < 0.00001.

Previously, we have observed that basal-like tumors with wild type copies of TP53 have elevated levels of CTL expression compared to those with one or more mutations of TP53 [43]. Since TP53 is a guardian of genomic stability, we generalized this idea here, examining whether tumors with regions of severe local genomic instability have differing lymphocyte infiltration patterns, using the complex arm-wise aberration index (CAAI) devised by Russnes et al. [44]. CAAI positive samples have many local gains and losses on at least one chromosomal arm and an association with estrogen receptor negativity, poor prognosis, and high tumor grade [44]. We found, when examining the METABRIC cohort, that CAAI positive samples had diminished B cell (*Wilcoxon rank sum test, p* = 2.53e^−5^), CTL (*p* = 3.62e^−10^), Th1 (*p* = 0.01) and Th2 (*p* = 2.50e^−11^) infiltration. This effect is markedly more pronounced in ER- tumors but still present in ER+ samples (B cell: *p* = 1.74e^−6^, CTL: *p* = 1.40e^−5^, Th1: *p* = 7.33e^−5^, Th2: *p* = 6.58e^−4^), and the predominantly ER- subtypes (Figure 6, Supplementary Figure 6), basal (*p* = 2.76e^−5^, *p* = 1.44e^−4^, *p* = 0.001, *p* = 0.004) and HER2 (*p* = 0.018, *p* = 0.004, *p* = 0.01, *p* = 0.014), and is not immediately evident when the experimentally defined METABRIC immune infiltration classes are used.

### Aromatase inhibition and increased infiltration

**Figure 7:**
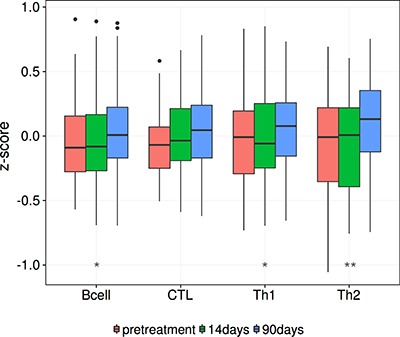
Lymphocyte infiltration increases over the course of Letrozole treatment This box plot shows the increase in lymphocyte infiltration over the course of Letrozole treatment. A Wilcoxon signed rank test was used to assess the significance of these changes between patients at pretreatment and 90 day time points. *P*-values are denoted by asterisks, *p < 0.05, **p < 0.01, ***p < 0.00001.

As a preliminary investigation into the relationship between common therapies and lymphocyte infiltration, we examined infiltration levels pre and 90 days into treatment with an aromatase inhibitor. Aromatase inhibitors are a viable treatment option for women with ER+ breast cancer, and we postulated that inducing a more negative ER phenotype would be marked by an increase in lymphocyte infiltration. We tested our hypothesis on a set of previously published data [45] containing gene expression for 58 breast biopsies taken at two time points, pre-treatment and 90 days into treatment with Letrozole. We found that the mean levels of B cell and the T helper subsets increase throughout treatment (Figure 7; *Wilcoxon rank signed test*, B cell: p=0.001, CTL: *p* = 0.086, Th1: *p* = 0.011, Th2: *p* = 1.05e^−4^). This initial insight further establishes the link between estrogen as a main determinant of lymphocyte infiltration.

## DISCUSSION

Immune infiltration is now widely recognized as an important component in the development and proliferation of cancer. Experimental methods quantifying the composition and abundance of lymphocyte infiltration within the tumor bed are typically time consuming, costly, and are not easy to perform at a large scale. Additionally, no existing method allows for retroactive absolute profiling while preserving the tumor sample. Given the plethora of existing breast cancer gene expression data, we devised a method that addresses these challenges, without prior knowledge about sample composition, which can be easily used to predict the immune landscape of a sample. We then applied this methodology to improve our understanding of lymphocyte infiltration in breast tissue.

Several other studies [16, 17, 29, 46–48] have also assessed lymphocyte infiltration by profiling gene expression microarrays. The majority of these methods estimate immune infiltration using unsupervised clustering and differential expression analysis of breast cancer cohorts. The remainder perform larger scale meta-analyses expanding immune marker genes using correlation across breast cancer samples. Unlike studies where clustering reveals enriched immune activity, our method does not first require the selection of differential expression patterns in a cohort. Additionally, unlike the correlation based approaches focused on specific studies, we use a very large compendium consisting of multiple human blood samples that are not limited to the breast or cancer, enabling us to robustly identify immune cell markers. This makes our methodology more generalizable to other disease datasets and biological questions, and prioritizes the signal driven by infiltrating lymphocytes, lending more confidence to our results.

Recent deconvolution based approaches [49, 50], such as CIBERSORT [51] have been used with some success profiling the levels of many types of immune cells. However, such approaches require knowledge of all cell types present in a mixture and output relative proportions. Furthermore, CIBERSORT requires purified immune cell expression data to construct their underlying LM22 reference matrix. To assess whether we can achieve similar performance without this potential data limitation we ran CIBERSORT on a small dataset of purified immune cell populations [52] that was not used in the construction of their immune reference matrix. We compared the identification of all overlapping immune cell types profiled in this dataset using our method with CIBERSORT's B cell (memory and naïve B cell) and CD4 (CD4 naïve, CD4 resting memory, and CD4 activated memory T cells) cell types. We found that, both our method and CIBERSORT could correctly label 2/2 of the Bcell samples and 4/4 of the CD4 samples with ours further correctly separating CD4 into the Th1 (2/2) and Th2 (2/2) subsets, making our proposed pipeline the only viable method when purified cell type data are not available.

Any computational method that makes a claim about the mixtures of cells in a sample, whether a differential expression, meta-analysis, or a deconvolution based approach, will suffer decreased performance when the cell types to distinguish are very similar. The lymphocytes we profiled here share many of the same surface markers, which can potentially degrade the quality of computationally derived markers. Additionally, using a large set of the resulting genes for our markers can potentially introduce some weaker markers into the analysis. This, however, is remedied as the large set of genes can robustly capture infiltration signal when one or more genes are missing from the sample profile (Supplementary Figure 2). Taken together, these *in silico* methods should be seen as exploratory lymphocyte assays that can be readily applied to existing and yet to come gene expression data and not a conclusive replacement for experimental approaches.

One of the most striking findings in our work is how drastically lymphocyte infiltration patterns vary depending on the status of the estrogen receptor. We observed this effect in both tumor and normal tissue, with ER- samples having, on average, higher levels of B cells, CTL, Th1, and Th2 infiltration than ER+ samples. Previous studies have also found strong immune signals in ER- breast tissue [16, 46, 53] that were weak or absent in ER+ tissue, and furthermore observed high CTL infiltration as a positive prognostic factor only in ER- samples [18, 21–23]. Interestingly, no other study has examined this link between ER activity in normal breast tissue. The correlation that we found between ER- and increased infiltration suggests that ER+ breast tissue might be more susceptible to tumor formation, and may be one reason that increased exposure to estrogen increases a woman's overall risk of breast cancer. Several studies [53–55] have linked estrogen to the proliferation of T regulatory (Treg) cells, which suppress B and T lymphocytes. Another recent study by Generali et al. [55] showed a reduction in Treg cells following treatment with the aromatase inhibitor, Letrozole. The inhibition of Treg cells may explain why we observed an increase in lymphocyte expression levels 90 days into treatment with Letrozole, and we hypothesize that Tregs contribute to the decreased infiltration we see in ER+ relative to ER- tumors.

It is still unclear what underlying mechanisms determine the extent of immune infiltration in ER- tumors. Previously, we uncovered one potential component when we observed that mutations in TP53 in ER- tumors are associated with a decrease in the level of CTL infiltration [43]. Here we more generally detect a similar phenomenon with CAAI status in ER- samples, those with low complex genomic instability have higher levels of B cell, CTL, Th1, and Th2 infiltration relative to samples with high instability (Figure 6, Supplementary Figure 6). This echoes the relationship Vollan et al. [56] has shown between high CAAI and poorer survival. A rapidly mutating tumor has the potential to adapt and evade the protective mechanisms of the immune system, yet also increase the distress signal and promote immune recruitment to the tumor site. This balance between potentially increasing and actively decreasing immune infiltration might be further influenced by the specific genomic rearrangement pattern the tumor exhibits [57]. CAAI measures a specific type of instability, namely “firestorms” where regions in close proximity are amplified on the same arm. Here we found that a reduction in beneficial immune recruitment occurs particularly with such complex mutations.

Overall, we found that CTL and Th1 infiltration are the main contributors to good outcome, and that the levels of these lymphocytes tend to increase together along with B cells and to a lesser extent with Th2 cells (Figure 2B, Figure 4). Through univariate survival analysis we observed that lymphocyte infiltration is prognostic only in ER- tumors, with Th1 and CTL infiltration having the most effect. Interestingly, conflicting observations on B cell infiltration and outcome exist, with some suggesting that B cell infiltration is either not functionally significant [14], promotes metastasis [13], and others finding B cells to be an important factor contributing to prolonged survival and long term remission [30, 47–48]. Here we found that elevated levels of B are weakly associated with good outcome in ER- partitions (Figure 4). However, since B cell infiltration correlates strongly with that of the other lymphocytes, including CTL cells, this finding could be confounded with a CTL, or a general infiltration response driven effect. Regardless, increased infiltration of the four lymphocytes we profiled here seem to have positive effects that are not entirely captured when using looking at all lymphocytes in aggregate (Supplementary Figure 5). Reducing immune checkpoints, particularly blocking the mediatory functions of other lymphocytes such as T-regulatory cells, may thus be a fruitful avenue for treatment of breast tumors regardless of estrogen receptor status.

With our methodology for capturing cell type specific signal we were able to leverage existing studies to systematically study immune infiltration without needing physical access to or knowledge of the potential cell composition of the samples. This enabled us to comprehensively examine immune infiltration patterns mentioned in previous studies, reconcile conflicting reports regarding association of B cell infiltration and outcome, and uncover novel biology such as the correlation between CTL and estrogen receptor negativity in normal breast tissue and the decrease in B cell, CTL, Th1, and Th2 infiltration with complex genomic instability. Our method is easily extendable and provides an accessible way to profile the immune landscape in future studies and can be extended to other cancer types.

## MATERIALS AND METHODS

### Generation of cell specific markers

Nanodissection (described formally in [31]) was applied on a large human blood compendium consisting of 2,171 publicly available samples (Supplementary Table 1). Standards of positive and negative example genes (of various “confidence” tiers) were constructed from significant Gene2MeSH (http://gene2mesh.ncibi.org) annotations for the four lymphocyte cell types and monocyte. For a given cell type, the corresponding Gene2MeSH genes were divided into unique genes and those that overlapped with other cell type gene sets. Unique genes corresponding to the cell type of interest were used as high-tiered positives, and those that were relevant but overlapping were used as low-tiered positives. All unique genes from the remaining three lymphocyte cell types and monocytes were used as negatives. Samples in the compendium were downloaded from GEO and processed from the raw CEL files, using RMA in “affy” R package [32] with default parameters [33]. The ranked output of each nanodissection run was reduced into a set of high quality marker genes based on the potential markers’ co-expression. To reduce the ranked list into the final signatures we took the top 100 predicted marker genes for each category (Supplementary Table 2). Our findings throughout remain robust to the specific cutoffs used to build these gene sets.

### Clinical data acquisition

Normalized gene expression microarrays for the METABRIC discovery (*N* = 803) and validation (*N* = 614) cohorts were obtained from the European Genome-phenome Archive (accession: EGAS00000000083). Herein we combined both of these partitions and treated them as one cohort. TP53 sequencing and categorization of lymphocyte infiltration by H&E staining is described in Silwal-Pandit et al. [35]. The gene expression data and corresponding annotations for the MicMa (*N* = 108), the set of reduction mammoplasty and prophylactic mastectomies (*N* = 24), and the Letrozole data (*N* = 58), were obtained from Gene Expression Omnibus (GEO) (accessions: GSE3985, GSE20437, GSE20181). The MicMa dataset was used directly and GSE20437 and GSE20181 were processed according to the Affy pipeline described in the above section. After processing all datasets were further z-score transformed across genes using the following formula:

$$z_{\text{gs}}=\frac{x_{\text{gs}}-\mu_{g}}{\sigma_{g}}$$

where *z_gs_* is the z-score for sample *s*, gene *g*, *x_gs_* is the normalized intensity for *g* in *s* and *μ_g_* and *σ_g_* are the mean and standard deviation of *g* across all samples in the cohort.

### Statistical analysis

A nonparametric Wilcoxon rank sum test, Wilcoxon signed rank test, or two-way ANOVA was used to derive *p*-values comparing lymphocyte scores as appropriate. To assess the relationship between the experimentally defined infiltration categories and the lymphocyte infiltration scores, we calculated correlation using Kendall's rank correlation between the infiltration classes (ranked according to their severity) and the infiltration scores per sample. When assessing robustness, 50 random genes were sampled without replacement 50 times. Gene Ontology enrichment was calculated using PANTHER [58] on a 2017 version of the GO tree. Reported *p*-values have been corrected for multiple hypothesis testing using Bonferroni. Univariate Kaplan-Meier estimates and accompanying log rank tests were calculated using the “survival” R package [36]. Correlations herein were calculated using the Pearson correlation coefficient. All *p*-values less than 0.05 were considered significant.

### Infiltration and ER activity scores

Clinical estimates of ER activity were taken from the publically deposited data. For the MicMa cohort ER activity was determined using monoclonal antibodies against ER, the details of which can be found in [39]. ER classification for METABRIC was calculated computationally using a Gaussian mixture model [38].

Scores measuring both immune infiltration and ER activity were calculated in the same way for a given sample, *s*, by averaging the z-score transformed expression of all *n* genes in the marker.

$$d_{s}=\frac{1}{n}\sum_{i=1}^{n}z_{\text{is}}$$

Genes in the marker sets not assayed in a given dataset were excluded from averaging. To calculate total lymphocyte infiltration we combined the genes in all four signatures into one before averaging.

Marker genes that capture ER activity were obtained from van't Veer et al. [37]. Increased activity of the estrogen receptor corresponds with higher ER activity scores. As a rule of thumb, ER activity scores less than zero correspond to ER- samples and scores greater than zero, ER+.

### Comparison with CIBERSORT

GSE3982, obtained and processed as described above without z-score normalization, was uploaded to the CIBSERSORT web interface and run with 100 permutations and quantile normalization disabled. Infiltration scores for the nanodissection markers were calculated as described in the previous sections. GSE3982 contained two samples for three cell types, B cell, Th1, Th2. Samples were assigned labels by taking the name of the highest scoring cell type for that sample. CIBERSORT's multiple B cell types (memory and naïve) were combined into one B cell label, as were the CD4 T cell types (CD4 naïve, CD4 memory resting, CD4 memory activated) for classification of the Th1 and Th2 samples.

## SUPPLEMENTARY MATERIALS FIGURES AND TABLES









## Abbreviations

ECM: extracellular matrix
CTL: CD8+ cytotoxic -T lymphocytes
Th1: CD4+ T helper 1 cells
Th2: CD4+ T helper 2 cells
TIL: tumor infiltrating lymphocytes
ER: estrogen receptor
CAAI: complex arm-wise aberration index
