# Supplementary material for: Data-driven analysis of immune infiltrate in a large cohort of breast cancer and its association with disease progression, ER activity, and genomic complexity
Source: Oncotarget. 2017 Jul 7;8(34):57121–33. doi: 10.18632/oncotarget.19078 (PMC5593630; doi:10.18632/oncotarget.19078)
Supplement: Supplementary file 1 [file oncotarget-08-57121-s001.pdf]

Data-driven analysis of immune infiltrate in a large cohort of breast cancer and its association with disease progression, ER activity, and genomic complexity

SUPPLEMENTARY MATERIALS

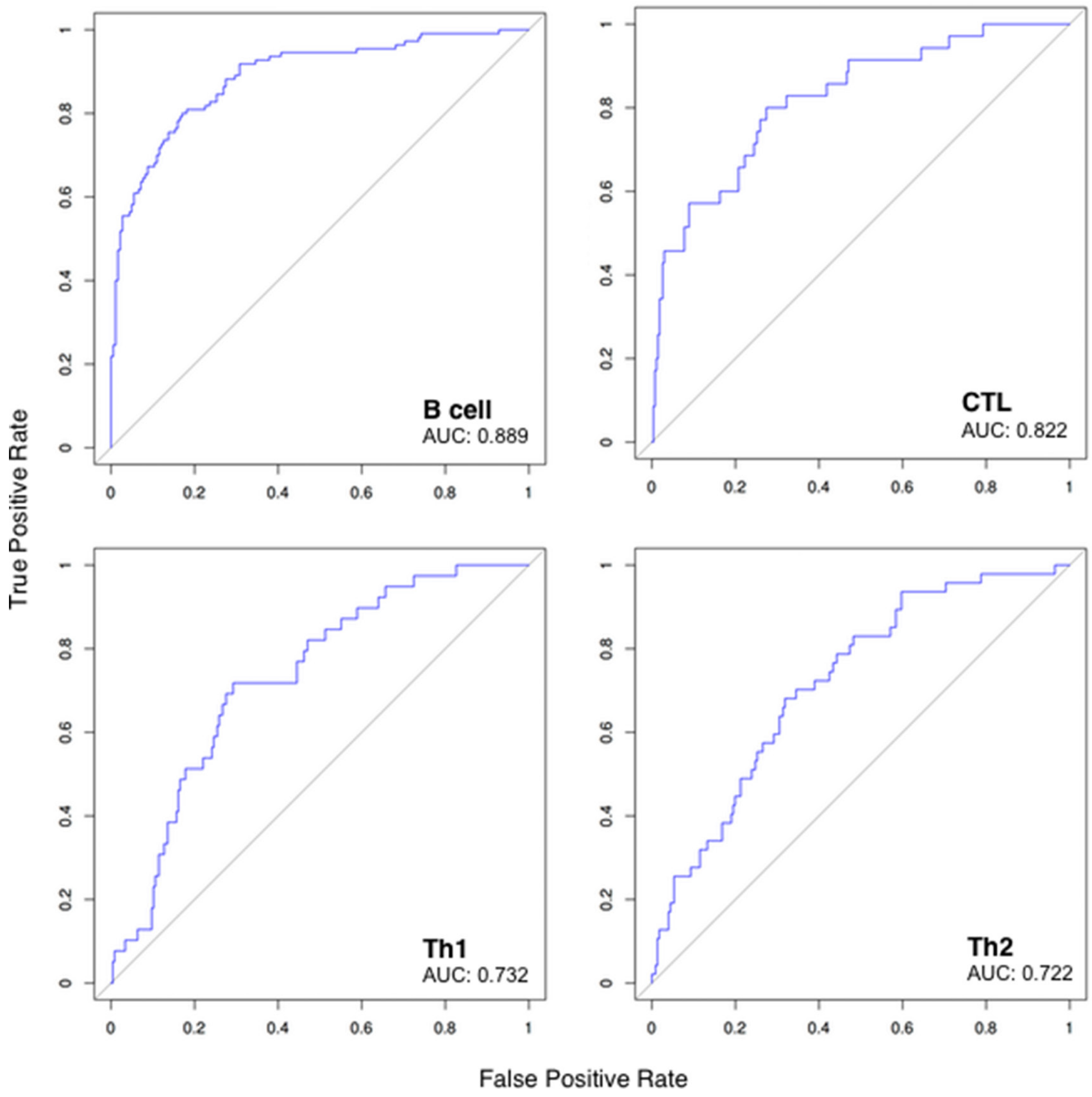

Supplementary Figure 1: Classification performance. ROC curves for each run of nanodissection.

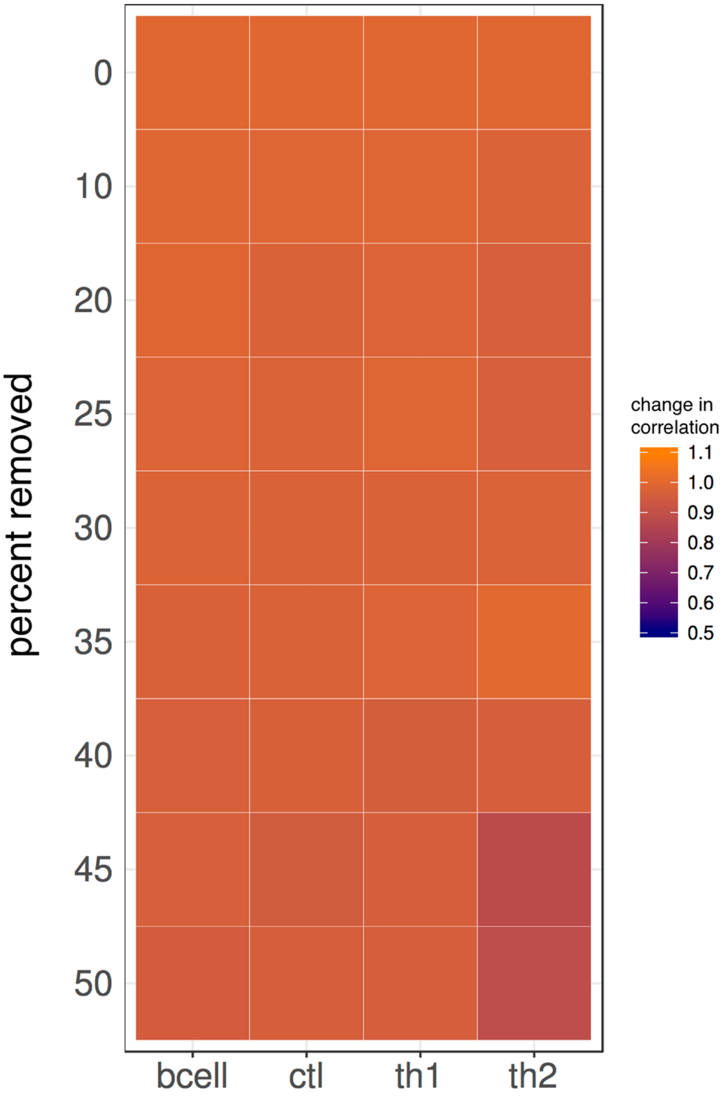

Supplementary Figure 2: Cell specific infiltration estimates can robustly recapitulate lymphocyte infiltration signal when some marker genes are missing or are not assayed. This figure shows the change in Kendall's Tau values relative to the correlation calculated with the full set of marker genes (no genes removed). Correlation is calculated between the ranked infiltration scores for a cell type and the experimentally derived infiltration categories (absent, mild, moderate, severe) across METABRIC. Colors indicate the ratio of the observed Kendall's Tau averaged over 50 iterations of random genes removed for each percentage. Only the Th2 marker shows a slight decrease in capturing this correlation when more than 45% of the genes are absent.

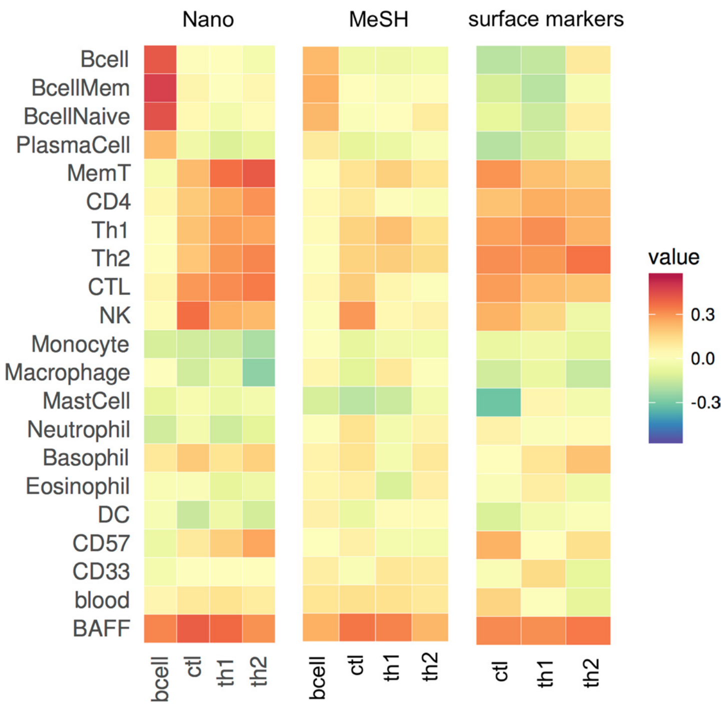

Supplementary Figure 3: Further evaluation of the nanodissection markers. The heatmap shows the average ssGSEA scores for the lymphocyte marker genes in gene expression datasets of purified immune cell types from Chtanova et al. [34], GSE3982, and GSE1133. The markers obtained using our nanodissection pipeline (left) have stronger enrichment in their corresponding cell types than the MeSH markers (middle) used as the gold standard or T cell surface markers (right) from BioCarta.

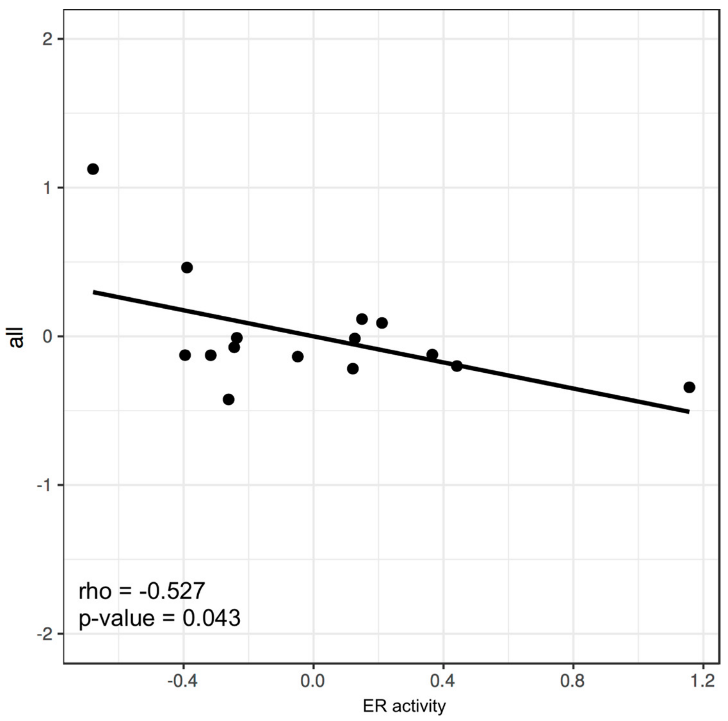

Supplementary Figure 4: Lymphocyte infiltration in a cohort of benign samples. ER activity and infiltration scores were calculated for $N = 15$ benign breast tumors [59]. In this cohort we also see a negative correlation between the level of lymphocyte infiltration and ER activity.

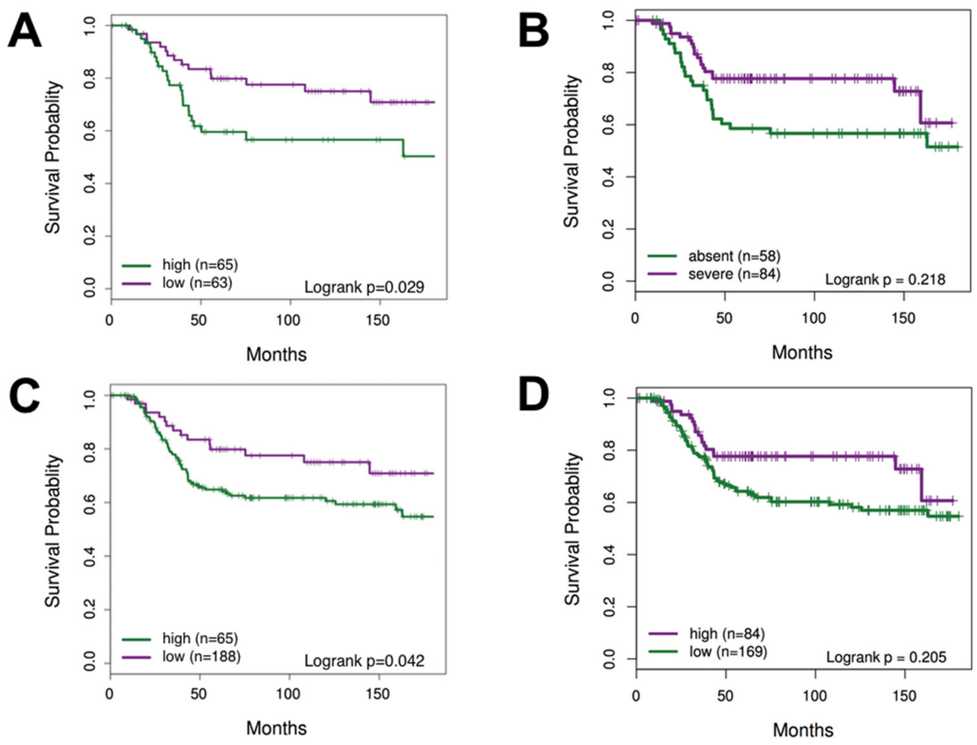

Supplementary Figure 5: Increased resolution of infiltration leads to better patient stratification. Partitioning ER- METABRIC samples using our expression of B cell, CTL, Th1 and Th2 lymphocytes (**A** and **C**) is compared with the lymphocyte staining derived annotations (**B** and **D**). In (A) we separate ER- patients using the lowest and the highest quartile of the infiltration scores and in (C) we compare the upper quartile (purple) with the remaining three (green). In both cases we are able to achieve a significant stratification, compared to using the two most extreme experimental annotations (B) or severe vs. absent, mild, and moderate (D) to stratify patients.

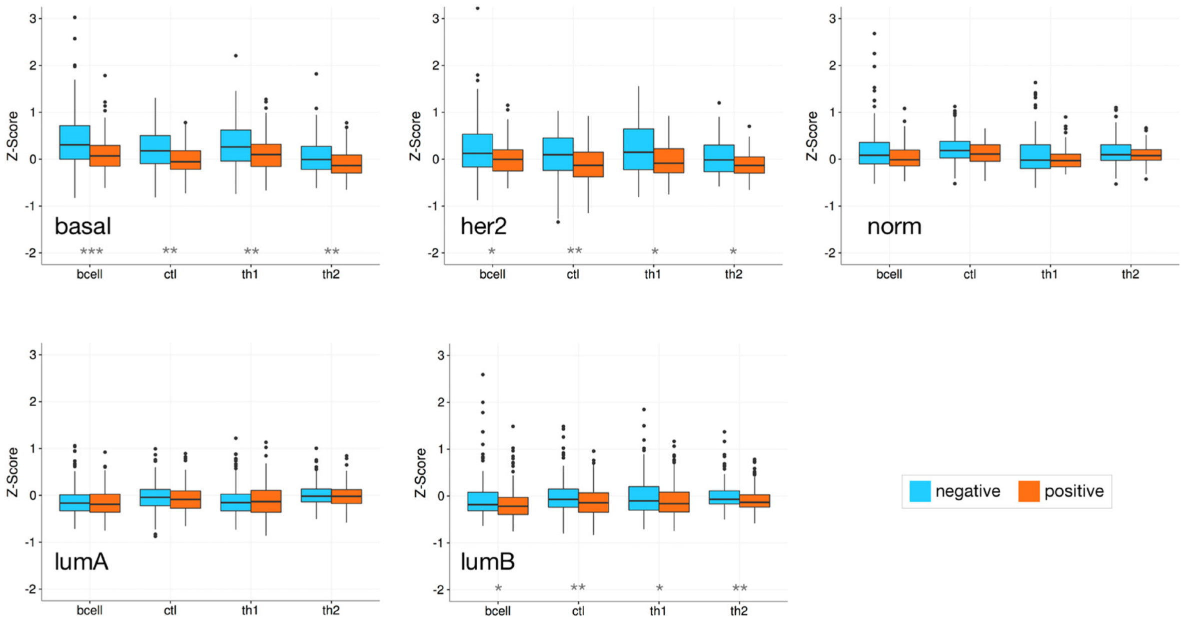

Supplementary Figure 6: Genomic complexity stratified by subtype. In all but luminal A and normal-like tumors, increased infiltration is associated with reduced genomic complexity, as measured by CAAI status. Asterisks correspond to p-values, *$p < 0.05$, **$p < 0.01$, ***$p < 0.00001$ calculated using the Wilcoxon rank sum test.

Supplementary Table 1: GEO samples used in the human blood compendium. See Supplementary_Table_1

Supplementary Table 2: Genes in the nanodissection markers. See Supplementary_Table_2

Supplementary Table 3: Significantly enriched GO terms for the resulting markers. See Supplementary_Table_3
